# The Effect of Different Temperatures on the Viability and Senescence of Plum Ovules (*Prunus domestica* L.)

**DOI:** 10.3390/plants13101359

**Published:** 2024-05-14

**Authors:** Milena Đorđević, Radosav Cerović, Mekjell Meland, Milica Fotirić Akšić

**Affiliations:** 1 Fruit Research Institute, Kralja Petra I/9, 32000 Čačak, Serbia; mdjordjevic@institut-cacak.org; 2 Innovation Centre of the Faculty of Technology and Metallurgy, University of Belgrade, Karnegijeva 4, 11120 Belgrade, Serbia; radosav.cerovic@gmail.com; 3 NIBIO Ullensvang, Norwegian Institute of Bioeconomy Research, Ullensvangvegen 1005, 5781 Lofthus, Norway; 4 Faculty of Agriculture, University of Belgrade, Nemanjina 6, 11080 Belgrade, Serbia

**Keywords:** stone fruit, female sporophyte, callose, degenerative processes

## Abstract

This paper reports on a study investigating the viability and senescence of plum ovules when exposed to different constant temperatures over two years. The research was conducted on the primary and secondary ovules of four plum cultivars: ‘Mallard’, ‘Edda’, ‘Jubileum’, and ‘Reeves’. The results show that the first indication of ovule viability loss was callose accumulation, which was detected using the fluorescent dye aniline blue. All cultivars had viable ovules, in different percentages, at 8 °C on the twelfth day after anthesis. However, at higher temperatures, distinct patterns emerged, indicating the adaptability of each cultivar at certain temperatures. The first indication of callose accumulation became visible at the chalazal pole. After anthesis, the ovule’s ability to remain viable gradually reduced, followed by callose deposition throughout the ovary. The cultivars ‘Edda’ and ‘Reeves’, from 6 days after anthesis onward, in both years, showed the highest percentage of nonviable ovules. In contrast, the ‘Jubileum’ cultivar demonstrated the highest percentage of viable ovules. The loss of viability of secondary ovules followed a similar pattern to that of the primary ovules in all cultivars. This research provides valuable insights into embryological processes, which can help in the following breeding programs, and to cultivate plum cultivars in Western Norway’s climate conditions.

## 1. Introduction

European plum (*Prunus domestica* L.) is mainly cultivated in the temperate regions of the Northern Hemisphere, but it can also grow well in cooler climates [1]. This fruit species is highly genetically diverse [2] and is used for various purposes, such as table consumption, drying into prunes, distilling, and processing multiple food products. European plum is a rich source of sugars, bioactive, antioxidant, and phenolic compounds, which can provide many health benefits to humans [3,4]. Furthermore, it is an essential fruit crop globally, with China being the largest yearly producer [5].

In Norway, plum production is mainly located at a latitude around 60° north in the fjord districts of the southwestern part and around lakes in the eastern part [1]. Short growing seasons and low summer and winter temperatures are some problems affecting plum growing. At the same time, some benefits include the long days, high global irradiation, delayed flowering, and a low risk of spring frost [6]. Plum-growing at this latitude is possible thanks to a warm gulf stream from the Atlantic Ocean [7]. The main cultivars are ‘Opal’ and ‘Victoria’, while ‘Jubileum’ and ‘Reeves’ have significant production areas [8]. All of the fruit produced is mainly used for fresh consumption.

The female sporophyte of plums consists of a monocarpellary pistil that contains two ovules. These ovules are attached to the placenta through a vascular bundle that runs through the funiculus and ends at the chalaza [9]. To produce a good fruit set, it is necessary to fertilize one of the ovules. Certain *Prunus* species have been found to have low fruit sets and EPP (effective pollination period, i.e., the period for which a flower retains its ability to give a fruit) due to unfavorable environmental conditions. The temperature of the air is a critical factor in the growth and development of fruit. Temperature fluctuations throughout the seasons can affect various biological processes. Studies have shown that changes in temperature patterns have impacted the growth of wild plants, fruit trees, and crops across 21 European countries [10]. In particular, high temperatures in the days or weeks after a full bloom (anthesis) can significantly affect the fruit set [11,12]. According to the Climate Model (CMIP6), Europe is expected to experience an increase in air temperature of 2 °C to 6 °C by the end of this century [13]. However, some experts argue that the timing and frequency of extreme temperatures, rather than an increase in average temperatures, have a more significant impact on fruit production [14]. These temperature changes represent challenges not only to plant productivity, but also to their geographic distribution. Heat stress at each stage of the reproductive process affects fruit trees and ultimately impacts fruit sets [15].

Various species of *Prunus* have been reported to be affected by high temperatures that can affect male gametophyte development, leading to reduced pollen viability, germinability, and pollen tube growth, both in vitro and in vivo [14,16,17,18,19,20]. The sporophytic anther wall, particularly the tapetum or the outer layers, can degenerate prematurely or in an untimely fashion due to the effects of high temperatures [21,22]. This degeneration can result in pollen sterility and anther dehiscence [23,24]. Earlier research disproved that female sporophytes and gametophytes were less sensitive to high temperatures than pollen [25]. However, several studies have suggested that temperature can impact the structures of female sporophytic and gametophytic tissue, leading to a decrease in stigmatic receptivity [26], shorter style length [11], changes in carbohydrate reserves [27], and accelerated ovule aging [28,29].

Callose, a cell wall component, is known for its high impermeability, rapid synthesis, and easy degradation [30]. Its production is triggered by wound healing, pathogen infection, and physiological strains [31]. As embryological data indicate, callose is temporarily deposited in a cell wall during normal reproductive processes [32]. However, extended callose deposition in ovules leads to their reduced viability [33,34].

The objective of this study was to establish the temperature threshold necessary to forecast the length of ovule viability at steady temperatures of 8 °C, 12 °C, and 16 °C for up to 12 days after anthesis in four cultivars of plum: ‘Mallard’, ‘Edda’, ‘Jubileum’, and ‘Reeves’, cultivated in Western Norway. These temperatures are commonly experienced during the plum blossoming period in a specific western Nordic climate. The study’s findings will aid in developing successful plum cultivation strategies by considering the impact of temperature on ovule viability, and also support future breeding programs.

## 2. Results

Like other species of the *Prunus* genus, the plum has two ovules in the ovary that remain the same size throughout the flowering period. One of these ovules is considered the primary ovule and remains in the ovary’s locule to continue growing, while the other is the secondary ovule.

### 2.1. Viability of Primary Ovules

**Figure 1:**
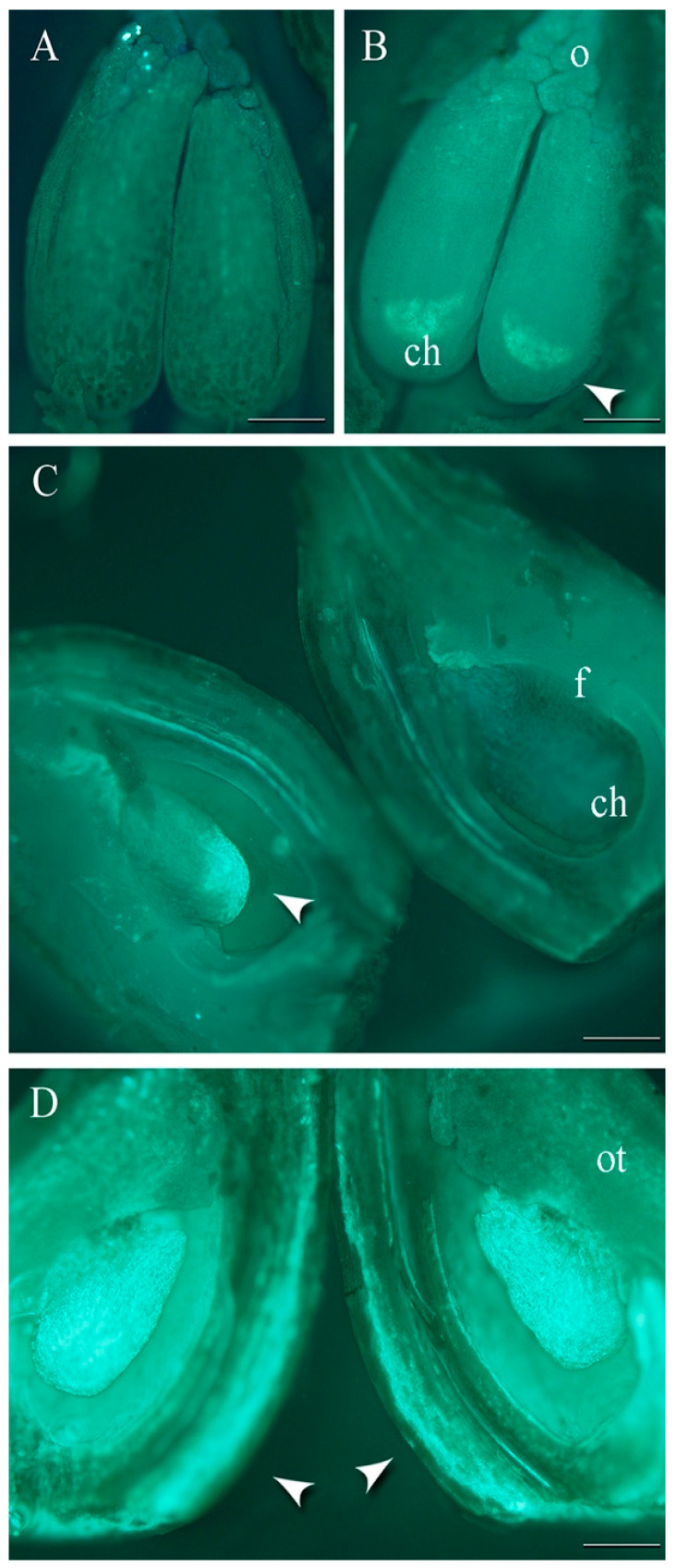
Stained ovule. (**A**) Ovaries at the anthesis. Callose fluorescence in the ovule and ovary tissue: (**B**) ‘Edda’ on the 2 DAA at 8 °C; (**C**) ‘Jubileum’ on the 12 DAA at 8 °C; (**D**) ‘Reeves’ on the 4 DAA at 16 °C. o—obturator, ch—chalaza, f—funiculus, ot—ovary tissue. Arrow—points to the accumulation of callose in the tissue of ovule and ovary; bars = 1 mm.

**Figure A1:**
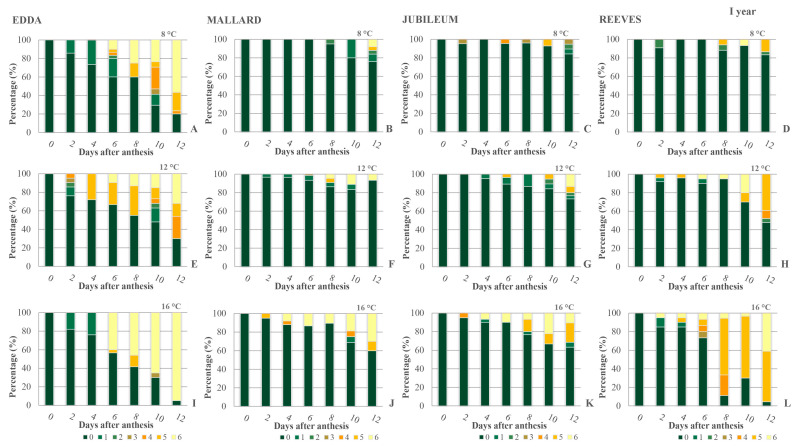
Observed stages of degeneration in the primary ovules during their senescences at different constant temperatures in the first year in the tested plum cultivars. (**A**–**D**) at 8 °C; (**E**–**H**) at 12 °C; (**I**–**L**) at 16 °C. Ovule fluorescence stages (0–6) are described in Section 4.3.

**Figure A2:**
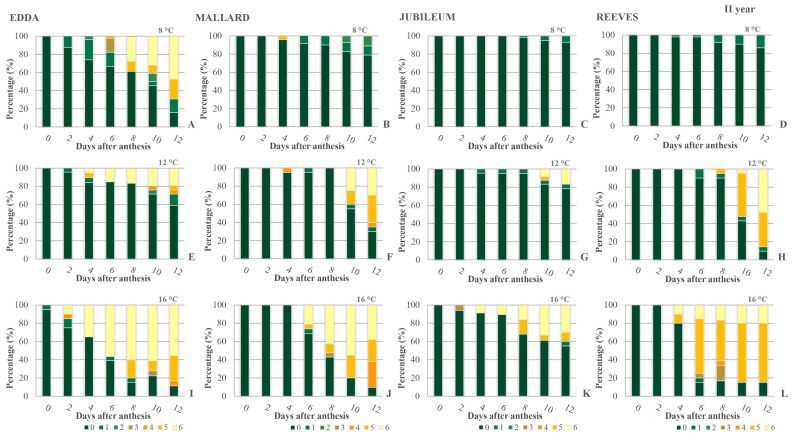
Observed stages of degeneration in the primary ovule during their senescence at different constant temperatures in the second year in the tested plum cultivars. (**A**–**D**) at 8 °C; (**E**–**H**) at 12 °C; (**I**–**L**) at 16 °C. Ovule fluorescence stages (0–6) are described in Section 4.3.

In both years, all tested cultivars showed no evidence of callose accumulation in the ovary at anthesis (Figure 1A, Appendix A Figure A1 and Figure A2). The cultivar ‘Edda’ had the lowest percentage of viable ovules in the days following pollination at 8 °C in both years. Only 20% of ovules were viable in the first year and 15% in the second year (Figure 1B and Appendix A Figure A1(A) and Figure A2(A)). During both years, the cultivar ‘Mallard’ had more than 80% viable ovules at 10 DAA, while ‘Jubileum’ and ‘Reeves’ showed the same results at 12 DAA (Figure 1C and Appendix A Figure A1(B–D) and Figure A2(B–D)).

The percentages of viable ovules of ‘Edda’ decreased at 12 °C in both years. At 12 DAA, the percentage of viable ovules was 30%, whereas in the second year, that percentage was over 50% on the same day. During the first year, at 12 °C, more than 60% of ovules were viable in ‘Reeves’, while in ‘Mallard’ and ‘Jubileum’, that percentage was around 80% on the same day. In the second year, on the same day, the percentage of viable ovules was lower, ranging from 42 to 55%, except for ‘Jubileum’, where over 80% of the ovules were viable (Appendix A Figure A1(E–H) and Figure A2(E–H)).

**Figure 2:**
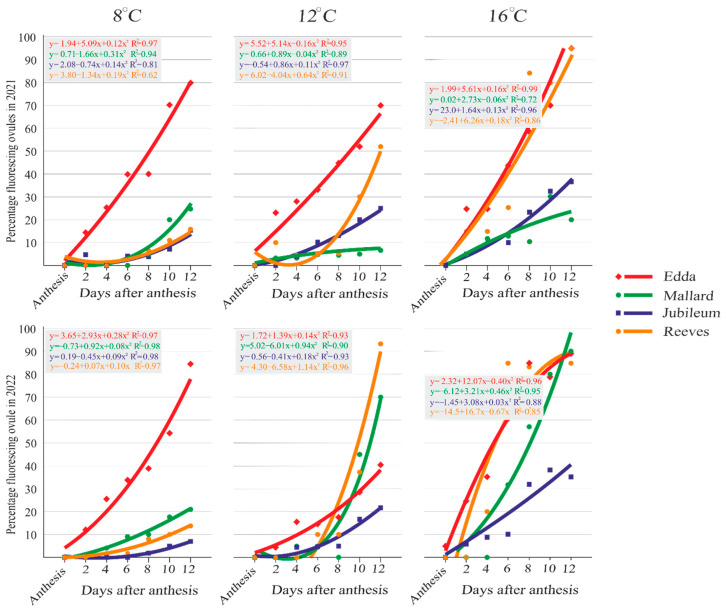
Regression analysis of fluorescing percentage in the primary ovules during the studied period in the tested plum cultivars. Each plot symbol in the regression line indicates the cumulative value of all stages of ovule degeneration observed in the fixation term of the cultivars shown in the legend. Equations and R^2^ values are located above or below the corresponding line.

The cultivars ‘Edda’ and ‘Reeves’ showed reduced ovule viability in both years when exposed to 16 °C (Figure 1D, Appendix A Figure A1(I,L) and Figure A2(I,L)). In cultivars ‘Jubileum’ in both years and ‘Mallard’ in the first year, a high percentage of ovule viability was observed at 16 °C (Appendix A Figure A1(J,K) and Figure A2(J,K)). Figure 2 depicts the regression line, which shows an upward trend in the percentage of ovules with fluorescence in the tested cultivars for several days after anthesis. Ovule degeneration was significantly associated with cultivars, temperatures, and the days after anthesis.

### 2.2. Callose Detection in Primary Ovules

Generally, the percentage of primary ovules with signs of fluorescence increased in the days following anthesis. Up to 4 DAA, most fluorescent ovules were at stages 1 or 2 in all cultivars at all tested temperatures. At a constant temperature of 8 °C, the callose at the chalazal part (stage 1) was observed earlier in the ovary of the cultivar ‘Edda’ at 2 DAA in both years, and in the other part of the ovary, it was detectable in the following days. All stages of fluorescence during ovule senescence with variation within cultivars and temperatures could be found at 6 DAA, while stages 5 and mostly 6 could be found at 12 DAA. The gradual progression of ovule fluorescence was most apparent in the cultivar ‘Edda’ at 8 °C in both years (2021 and 2022: R^2^ = 0.97). This cultivar’s ovule fluorescence at stage 6 varied from 9.99% at 6 DAA to 56.61% at 12 DAA in 2021, and from 27.55% at 8 DAA to 47.38% at 12 DAA in 2022. The highest percentage of ovule fluorescence at stage 6 at 12 °C was observed in ‘Edda’ in the first year (from 9.32% at 6 DAA to 32% at 12 DAA), while this stage in the second year at 12 DAA was found in 47.6% of ‘Reeves’ ovules. At stage 6, ovule fluorescence was observed at 16 °C in cultivar ‘Edda’, the highest percentage in both years. In 2021, it ranged from 39.96% at 6 DAA to 95% at 12 DAA. In 2022, it ranged from 10% at 2 DAA to 55.5% at 12 DAA. During both years at 16 °C, the ‘Jubileum’ cultivar had ovule fluorescence lower than 39% at stage 6. The values of the multiple correlation coefficients (R), ranging from 0.62 to 0.99, show that these stages of ovule degeneration were highly correlated with the fixation term, in both years, for all studied cultivars.

### 2.3. Secondary Ovules—Viability and Callose Detection

**Figure A3:**
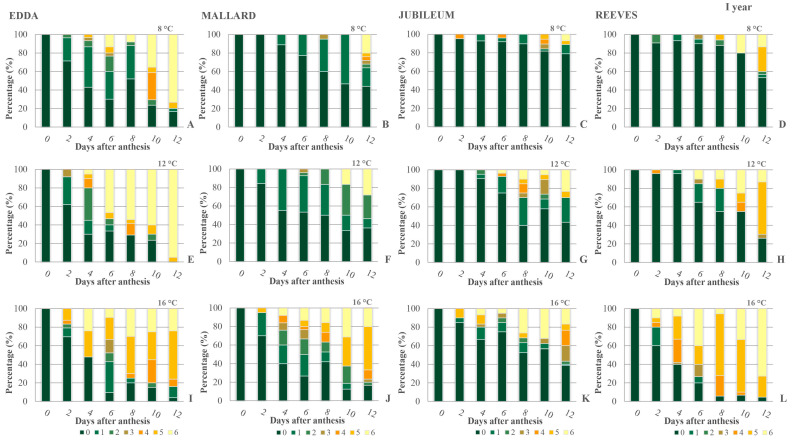
Observed stages of degeneration in the secondary ovule during their senescence at different constant temperatures in the first year in the tested plum cultivars. (**A**–**D**) at 8 °C; (**E**–**H**) at 12 °C; (**I**–**L**) at 16 °C. Ovule fluorescence stages (0–6) are described in Section 4.3.

**Figure A4:**
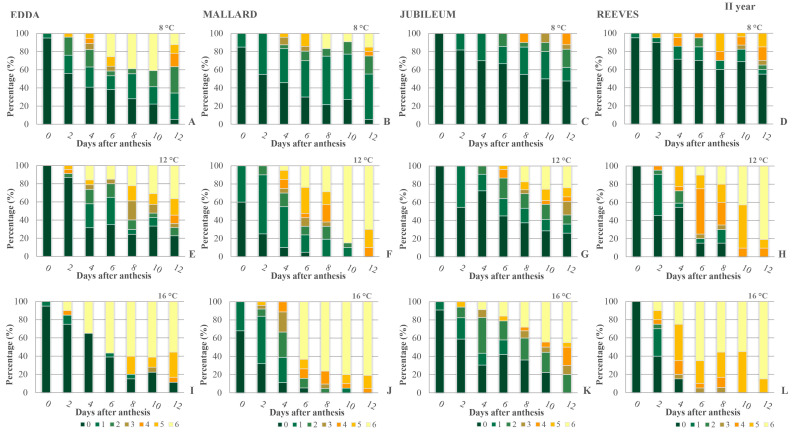
Observed stages of degeneration in the secondary ovule during their senescence at different constant temperatures in the second year in the tested plum cultivars. (**A**–**D**) at 8 °C; (**E**–**H**) at 12 °C; (**I**–**L**) at 16 °C. Ovule fluorescence stages (0–6) are described in Section 4.3.

All cultivars showed no fluorescence at the anthesis of the secondary ovules (Appendix A Figure A3 and Figure A4). The secondary ovules followed the events in the primary ovules. In the days following anthesis in the first year in the cultivar ‘Edda’, a drop in ovules without fluorescence was evident at 8 °C at 12 DAA (16%), whereas the cultivars ‘Mallard’, ‘Reeves’, and ‘Jubileum’ showed a high percentage of ovules without fluorescence (44%, 53%, and 93%, resp.). The percentages of viable ovules in the ‘Jubileum’ and ‘Reeves’ were around 50% at 8 °C on the 12 DAA in the second year. In both years, at 12 °C and 16 °C, a significantly lower number of ovules without signs of fluorescence were observed (Appendix A Figure A3(E–L) and Figure A4(A–L)).

**Figure 3:**
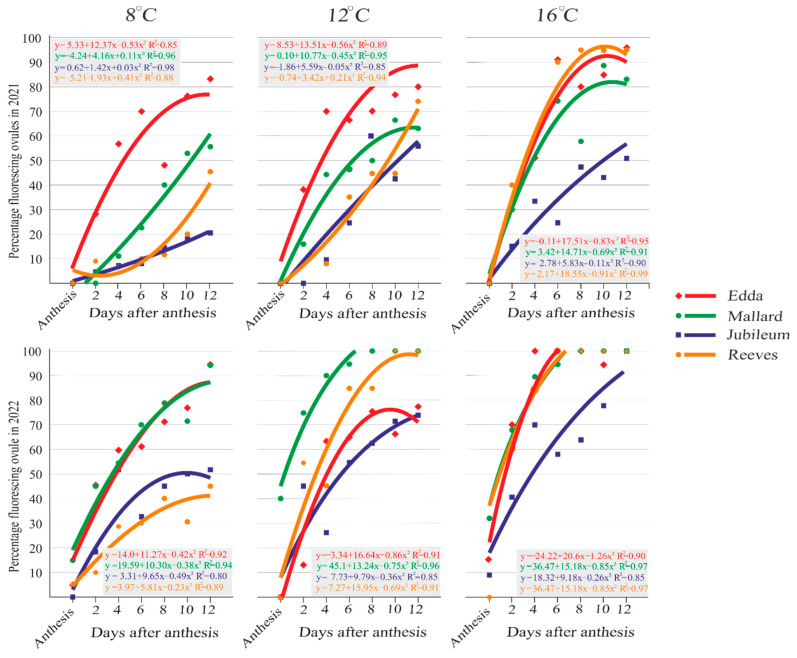
Regression analysis of fluorescing percentage in the secondary ovules during the period analyzed in the tested plum cultivars. Each plot symbol in the regression line indicates the cumulative value of all stages of ovule degeneration observed in the fixation term of the cultivars shown in the legend. Equations and R^2^ values are located above or below the corresponding line.

The occurrence of stage 1 of ovule degeneration was determined at 2 DAA, whereas on the other days, all other stages of fluorescent ovules were present at different percentages. The percentages of ovules with fluorescence in the cultivars tested on the days following anthesis manifested a type of parabola. The correlation coefficient index was between 0.80 and 0.99, which indicates that the analyzed factors are highly intercorrelated (Figure 3).

The fastest growth trend in the number of fluorescent ovules in the first year at the days after anthesis at 8 °C was found in the cultivar ‘Edda’, while at 12 °C and 16 °C it was matched by ‘Reeves’. In the second year at 8 °C, the fastest trend was seen in ‘Edda’, while at 12 °C it was seen in ‘Mallard’ and ‘Reeves’. At 16 °C, almost all the analyzed cultivars showed the same growth trend for ovule degeneration with strong fluorescence, except that it was slightly weaker in the cultivar ‘Jubileum’.

## 3. Discussion

We are currently investigating how temperature impacts the viability of plum ovules. Given that air temperatures are expected to increase due to global warming, we use various constant temperatures from anthesis up to 12 DAA. Temperature changes can significantly affect biological processes in fruits, which may result in poor fruit sets. High air temperature has been shown to affect various reproductive aspects, such as stigma receptivity, pollen vitality, pollen tube growth, and ovule longevity [35,36,37,38,39], although many parts are still unknown.

In our research, the lowest constant temperature used was 8 °C. During both years of the investigation, the observed viability of primary ovules in the tested cultivars remained the same at this temperature. Among the tested cultivars, ‘Jubileum’ and ‘Reeves’ showed a high percentage of ovule viability on 12 DAA, while the cultivar ‘Edda’ showed a high rate of ovule viability on 8 DAA. This finding is consistent with Đorđević’s research [40], which suggests that the European plum has long ovule viability in natural conditions, lasting up to 10 days from the moment of full flowering.

According to the study, when the temperature was increased by 4 ºC and 8 ºC in the tested cultivars, the period during which the primary ovule remained viable was reduced, resulting in a shorter time to 8 DAA and 10 DAA. Among these cultivars, the fastest loss of ovule viability was observed in ‘Edda’ at these temperatures, except in the second year at 12 °C, where a relatively high percentage of viable ovules was observed up to 12 DAA. These results are consistent with previous research by Cerović and Ružić [16] and Cerović et al. [29], which indicates that cherries and plums lose ovule viability as temperatures increase. A study of five European plums found that high ovule viability (80–100%) was observed when pistils were exposed to 5 °C, 10 °C, and 15 °C during the first ten days after full flowering. However, all tested plum cultivars showed a drastic drop in ovule viability at a constant temperature of 20 °C. Previous research suggests that pollen tubes do not enter ovules with intense fluorescence, as they are considered non-viable or senescent [41]. However, in the majority of cases for sour cherry, under the controlled conditions, and in a few cases under field conditions for sweet cherry, when primary ovules were fluorescent, a clear penetration of pollen tubes was observed [16,39]. The lower percentage of fluorescent primary ovules in open pollination compared to cross-pollinated cultivars may be due to the absence of flower emasculation [14,42].

The exact factors that cause the degeneration of one of the two ovules, a natural process in the flowers of *Prunus* species, are unclear. In Japanese plum flowers, the primary and secondary ovules are the same size when opening, but each embryo sac has different developmental stages [43]. The sizes of both degenerated and vital ovules may be influenced by adverse environmental conditions before flowering, according to Guerra et al. [42]. In some European plums, the secondary ovule atrophies as soon as full bloom begins [29]. Previous studies have shown that the first sign of secondary ovule degeneration is the appearance of callose at the chalazal pole of the ovules [34,35,42]. Our research has observed the first stages of fluorescence in secondary ovules at all constant temperatures in both years on the 2 DAA. In cherries, most ovules become senescent at 5 DAA at 5 °C, while at 20 °C, this period is shorter [36].

Several studies have demonstrated the crucial role played by callose in various processes of plant development and the plant’s response to different biotic and abiotic stresses [31,44,45,46]. The occurrence of callose at the chalazal pole in tested plum cultivars is a result of increased ethylene concentration, according to Gonkiewicz and Nosal [46]. Whether ethylene stimulates callose production or inhibits endosperm development, leading to callose synthesis, is uncertain. According to Pimienta and Polito [33] and Rodrigo and Herrero [34], the deposition of callose in the ovule blocks the flow of assimilates and depletes starch. However, it is still unclear whether the synthesis and deposition of callose in the chalazal pole is responsible for the degeneration of the ovule. Recent findings in unpollinated flowers of *Arabidopsis* indicate that ovule integuments degenerate before the gametophytes, and that the later degeneration of gametophyte cells occurs via passive starvation, a consequence of integument degeneration [47]. These findings are consistent with previous research conducted on the senescence of ovules in *Prunus* species [16,35].

The annual variation in ovule senescence suggests that genetic factors, adverse environmental conditions, and the nutritional stage of the flower influence it [43]. In our research, the cultivar specificity affected the expression, i.e., its sensitivity to certain constant temperatures. The tested cultivar’s genetic backgrounds could be one reason for this behavior. All cultivars were from cooler geographic regions: ‘Mallard’ from England, ‘Edda’ from Norway, ‘Jubileum’ from Sweden, and ‘Reeves’ from Canada. The cultivars ‘Jubileum’, ‘Mallard’, and ‘Reeves’ have shown more resistance to temperature stress. The highest percentage of viable ovules was observed with the ‘Jubileum’ cultivar at all temperatures. This cultivar exhibited the highest percentage of viable and fertilized embryo sacs and fruit sets, whereas the lowest was observed in ‘Reeves’, as Cerović et al. [48] reported. Previous research by Fotirić Akšić et al. [1] indicated that ‘Jubileum’ has a longer effective pollination period of five days, in contrast to ‘Edda’, ‘Mallard’, and ‘Reeves’, where it lasts only three days. The authors suggested that temperature differences during the flowering and post-pollination periods could contribute to the fruit-set discrepancy.

## 4. Materials and Methods

### 4.1. Plant Material

This research was conducted during 2021 and 2022 with plum branches from the orchard located in Ullensvang, Hardanger region, western Norway (15 m a.s.l., 60.318655 N, 6.652948 E, with a mean annual temperature of 7.6 °C and 1705 mm precipitation/year, and 12.3 °C and 638 mm for the growing season). For the study of the effects of different constant temperatures on ovule longevity and senescence, branches from the 8-year-old plum trees of the cultivars ‘Mallard’, ‘Edda’, ‘Jubileum’, and ‘Reeves’ were used. All trees were grafted on the ‘St. Julien A’ rootstock. Branches taken from the periphery of the canopy of the selected trees mostly contained flowers in the same phenological phase (BBCH59; Meier [49]) (flowers in the earlier or later phenophase on branches were removed).

### 4.2. Experimental Designs, Staining, and Sample Preparation

In the lab, cut branches were placed in jars filled with water. When the branches reached the complete flowering phase (BBCH61), they were transferred to chambers with three different constant temperature regimes (8 °C, 12 °C, and 16 °C) and distinct light cycle regimes. For each cultivar, 50 pistils (flowers) were collected every other day, starting from the day of full flowering (day 0) and continuing until the 12th day. The pistils were fixed using FPA fixative (70% ethanol:propionic acid:formaldehyde, 90:5:5, *v*/*v*) and refrigerated at +4 °C. The water in the glasses containing the branches was replaced daily until the fixation process was completed. The viability of ovules was assessed using the fluorescent microscopic method described by Anvari and Stösser [50]. The primary and secondary ovules were observed under UV light (blue filter) using an Olympus BX61 fluorescence microscope and DP70 camera. A total of 1440 ovules were analyzed, including 720 primary and 720 secondary ovules.

### 4.3. The Stages of Ovule Fluorescence during Its Senescence

**Figure 4:**
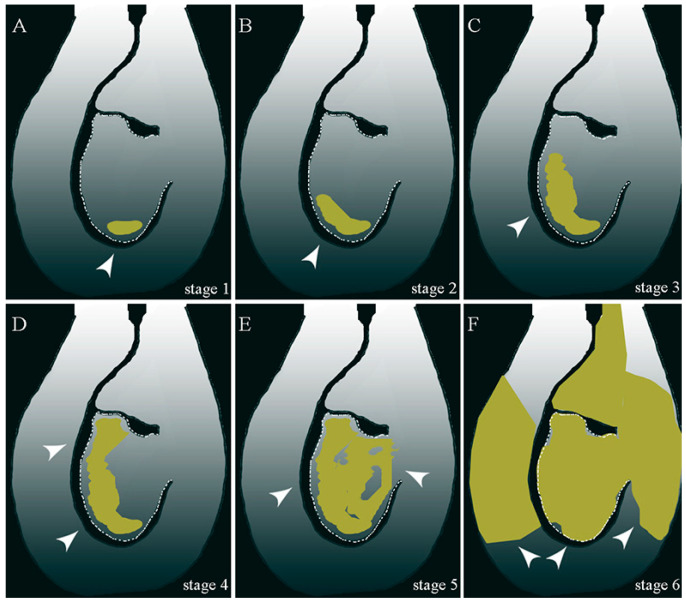
Scheme of ovule fluorescence/callose accumulation stages (yellow-golden area). The arrow points to fluorescence/callose accumulation in the ovule and ovary tissues.

At anthesis, due to the lack of fluorescence, ovules were indicated as fully viable (0 stage). In the days following anthesis, we categorized the ovary into six stages based on the varying intensity of aniline blue staining in different regions. The ovary’s first stage showed fluorescence in the chalaza pole (Figure 4). The fluorescence spread laterally in the second stage, mainly to the side opposite the funiculus. During stage 3, fluorescence spread towards the micropylar end. The micropylar end started to fluoresce in stage 4, and during stage 5, weak fluorescence could be observed in the whole ovule. In stage 6, strong fluorescence was visible in the entire ovule as well as the tissue of the ovary. To determine the percentages of ovules with and without fluorescence, a study was conducted on 30 flowers, considering the cultivars, temperatures, and fixation periods.

### 4.4. Statistical Data

Stats Blue online statistical software was used to obtain regression analyses and the correlation coefficient (R^2^) between ovules displaying fluorescence and the days after anthesis.

## 5. Conclusions

Depending on their genotype, it was concluded that different plum cultivars have varying reactions to constant temperatures during the first 12 days after full flowering. The tested plums had the highest percentage and longest ovule viability at the lowest constant temperature, reflecting their origin. On the other hand, the results obtained on ovule viability at higher temperatures may be related to the weaker/better adaptability of the genotypes. Since temperatures tend to rise during the flowering period, this reproductive parameter could be used to predict the reactions of these cultivars, taking into account the specific climatic conditions of western Norway. Besides supporting plum production in cooler climatic conditions by planting orchards with cultivars having high potential fertilization success and fruit sets, the results of this study can influence future breeding programs to consider using cultivars with longer longevity of ovules.

## Data Availability

Data are contained within the article.
